# Seven ways to get a grip on implementing Competency-Based Medical Education at the program level

**DOI:** 10.36834/cmej.68221

**Published:** 2020-09-23

**Authors:** JD Dagnone, D Taylor, A Acker, M Bouchard, S Chamberlain, P DeJong, A Dos-Santos, M Fleming, AK Hall, M Jaeger, S Mann, J Trier, L McEwen

**Affiliations:** 1 Queens University, Ontario, Canada

## Abstract

Competency-based medical education (CBME) curricula are becoming increasingly common in graduate medical education. Put simply, CBME is focused on educational outcomes, is independent of methods and time, and is composed of achievable competencies.^1^ In spite of widespread uptake, there remains much to learn about implementing CBME at the program level. Leveraging the collective experience of program leaders at Queen’s University, where CBME simultaneously launched across 29 specialty programs in 2017, this paper leverages change management theory to provide a short summary of how program leaders can navigate the successful preparation, launch, and initial implementation of CBME within their residency programs.

## Abstract (fr)

Les programmes de formation médicale fondée sur les compétences (FMFC) sont de plus en plus répandus dans les études supérieures en médecine. En termes simples, la FMFC est centrée sur les résultats scolaires, elle est indépendante des méthodes et du temps, et est constituée de compétences réalisables.^1^ Malgré cette adoption généralisée, il reste encore beaucoup à apprendre sur la mise en œuvre de la FMFC au niveau des programmes. Tirant profit de l’expérience collective des responsables de programmes à l’Université Queen, où la FMFC a été lancée simultanément dans 29 programmes de spécialité en 2017,le présent article s’appuie sur la théorie de la gestion du changement pour produire un court résumé de la manière dont les responsables de programmes peuvent gérer avec succès la préparation, le lancement et la mise en œuvre initiale de la FMFC au sein de leurs programmes de résidence.

## Introduction

Worldwide, competency-based medical education (CBME) curricular models are now increasingly common.^2^^,^^3^ However, those implementing CBME at the program level still struggle to know how best to do so.^4^ Put simply, CBME is focused on educational outcomes, is independent of methods and time, and is composed of achievable competencies.^1^ Bridging theory to practice is a challenging task, rife with potential pitfalls and misguided intentions.^5^ The wrong implementation strategy may result in stakeholder resistance, poor curricular alignment, information technology (IT) platform failure and program leader burn-out. Oversights and difficult conditions will surely be encountered if the processes driving change are rigid, non-iterative, and top-down, or neglect important support structures and personnel essential to the overall functioning of a program. Proceeding prematurely and/or unprepared puts a program at risk during the implementation process. Luckily, this need not be the case.

In 2017, 29 specialty programs at Queen’s University in Kingston, Canada launched CBME curricula simultaneously, uniquely positioning its program leaders to offer insights to inform others’ efforts in their implementation of Competency by Design (CBD) across Canada.^6^^,^^7^ Through their lived experiences, this cohesive group of postgraduate medical education leaders negotiated a shared understanding of CBME theory and worked collaboratively to navigate the institution-wide transition during the first years of implementation. This collective experience from our university context is shared in a series of tips, adapted from Kotter’s 8 step change model,^8^ about how to be successful when implementing CBME at the program level.

## How to get a grip on CBME within your program

### 1. Create a sense of urgency

Creating a sense of urgency by raising awareness about an existing problem and helping people see a possible solution is the first step in a change management process.^8^ In 2015, a 3-day program leader workshop was held at Queen’s University by the central CBME team, more than two years prior to the institutional 2017 launch date, to communicate the plans of the transformative change process and ignite early developments at the program level. This was immediately followed by messaging across all departments, divisions, and programs so that all stakeholders were aware that a new curricular model would be launched across all specialty programs within postgraduate medical education. Numerous stakeholder meetings and townhalls were held across the institution and division and department chairs met with program education leaders to begin steps to raise immediate awareness of CBME at the program level.

Early on, education leaders leveraged multiple opportunities within their programs at grand rounds, departmental meetings, education rounds, and using blogs and online resources to communicate that a large-scale transformative change was underway. It was explicitly communicated that this change would affect everyone – leaders, frontline faculty, trainees, program administrators, hospital partners, and patients.

### 2. Build a strong coalition

Since clinical education happens in a complex, resource-demanding context blending patient care, education, and research, it is important to think broadly about the composition of this coalition. According to Kotter’s model, identifying a team of influential people with a range of skills and experience to help champion change must be a priority.^8^ Ultimately, this coalition helps spread the change message, delegates tasks and ensures there is support for the coming change. After all, introducing CBME should be conceptualized as departmental change, not merely curricular renewal. As an early strategic priority, education leaders must become effective coalition builders—establishing strong partnerships with those who influence resource allocation decisions. Fostering mutually supportive relationships with department (and division) leaders, departmental resource committees, dean’s office personnel, and hospital leadership are essential early steps in the process. At a minimum, this means setting up regular meetings with departmental leaders to continually message the importance of investing in the change. This is not simply about financial support; these individuals and groups can facilitate necessary access to faculty time, administrative support, and avenues of communication.

### 3. Nurture a shared vision for change

Once the change coalition is in place, a concise, comprehensive, but accessible message must be developed that will inspire action.^8^ Determining the values that are central to CBME curricular reform and designing a plan for change are fundamental components of creating this unifying vision. Operationalizing CBME is disruptive and initially threatens the status quo of all involved. Adopting the right change management strategy helps address challenges related to stakeholder engagement, information sharing, and ultimately dealing with resistance.^9^ Early preparation involves not only information sharing about the benefits of CBME curricular reform, but also invites co-production with stakeholders about how best to operationalize features of these new curricula.

It is common to encounter conscientious objectors, masked as resistors, who perhaps don’t fully understand what is happening, need to be convinced of a need for change, or fear change in general.^10^ This initial resistance is mitigated by exploring the rationale for change, how CBME can assist in this pursuit, and underlining the iterative process of curricular reform. Early and ongoing stakeholder engagement supports individuals to adopt the required transformational shift. Nurturing ongoing conversations, debate, and discussions across all stakeholder groups (e.g., education leaders, administrative staff, faculty, residents) builds momentum of the CBME project and stimulates the emergence of collaborative solutions.

### 4. Invest in your existing program administrative assistants

Implementing CBME significantly expands the responsibility and complexity of the program administrator (PA) role. PAs will manage a larger flow of assessment data and reports, coordinate the sequence of meetings and reporting demanded in CBME, manage and direct faculty and residents engaged in the assessment program (academic advisors and competence committee members), implement an expanded, reliable, and closed loop communication strategy, and serve as a super-user of the electronic assessment platform. Success demands that PAs go beyond the ‘*what to do’* and have a clear understanding of the *why* and *how* of CBME program design.

Promoting regular collaboration across program administrators to form community networks also fosters the sharing of early lessons learned and resources developed to address shared needs. Such activities can reduce feelings of isolation and serve to distribute the burden of resource development when common needs are identified. Although many resources will be program-specific (e.g., EPAs, assessment tools, etc.), ideas gleaned from one program can be customized to the context-specificity of others. For example, adapting context-specific training materials for frontline faculty – including regional faculty (e.g., CBME assessor guides, EPA posters) and learners (e.g., CBME resident survival guide) can be very effective.

### 5. Pilot, pilot, pilot

Change theory cautions about the challenge of maintaining momentum over the long-term and advocates for the setting of short-term goals to help offset this risk.^8^ Piloting components of a new CBME program is a low-risk, effective way to field-test with minimal consequences. These types of activities engage faculty and learners in the change process and promote acceptance of change.^11^ For example, piloting assessment tools provides faculty the opportunity to experience their ease of use and promotes learners’ acceptance, ultimately lessening the enormity of change, and needed assessment tool modifications such as length of assessment, format, entrustment scales and electronic delivery methods prior to the official CBME launch date and initial implementation period. Such opportunities can also identify challenges, inform refinements, and promote feelings of ownership, but also offer everyone the chance to gain familiarity with the electronic platform before full implementation. This provides short-term wins that help motivate all involved.

There are many other examples of short-term wins to maintain momentum that should be celebrated within programs, such as new education champions recruited to the program, trainees interested in helping with CBME implementation, successful funding grants, academic projects and scholarship that stems from the CBME change, and positive developments in IT platform functionality.

### 6. Sustain longitudinal stakeholder development

Acknowledging the significant investment required to launch CBME, development initiatives must be maintained for program leaders, frontline faculty, and residents post-launch.^12^ This is ideally tailored to address ongoing stakeholder needs. For example, program leaders benefit from learning about strategies for leading and optimizing change efforts, especially in relation to shifting program culture.^13^ Providing opportunities for this group to share their challenges and useful strategies in a supportive environment fosters cross-pollination and provides emotional support and practical suggestions about navigating the inevitable implementation dip.

The long- term success also requires resident trainees to take an active role in their learning in CBME.^14^ While frontline faculty need orientation to entrustable professional activities (EPAs) and new assessment tools, residents also need to understand what is expected of them. As assessment paradigms shift, residents will need to move from simply becoming comfortable asking for EPA assessments on a daily basis to actively seeking out appropriate learning opportunities in order to fulfill their clinical and non-clinical experience requirements. Building on the cultural change necessitated by residents becoming the primary drivers of their assessment experiences, trainees can be encouraged to take progressively greater responsibility for identifying and addressing their learning needs.

Lastly, CBME is associated with a marked increase in assessment data collection, interpretation, and reporting. To support learning, coaching, progress and promotion decision-making, and appeals policies, programs must standardize these processes. Developing templates for learning plans, academic advisor reporting, and competence committee reporting are key steps towards success and consistency in these areas.

### 7. Prioritize program evaluation early on

Finally, Kotter ‘s change model highlights the need to integrate the change into the organizational culture by making it visible, continuing to support it, and publicly valuing key contributors.^8^ Documenting the shift to CBME via program evaluation is critical for understanding impact, understanding losses, and refining implementation efforts. With each program-level implementation, evaluation processes must be utilized to gather timely, formative feedback about whether the CBME program is being implemented as intended, uncover unintended outcomes, and identify iterative adaptations that need to occur. Multiple information sources inform such efforts, including for example, interviews, focus groups, stakeholder surveys, and data mining (e.g., assessment completion rates). With this knowledge in-hand, CBME implementation is improved in response to stakeholder experience informing iterative change, which also helps to inform others in their implementation efforts through dissemination of the evaluation results.^6^

### Conclusion

There are many challenges associated with implementing CBME at the program level. Bridging theory to practice brings with it many potential wrong turns and slips and requires explicit strategic planning to ensure success. Leveraging change management theory such as Kotter’s 8-Step Change Model^8^ and implementing it within our specific context promotes a better understanding of implementing CBME at the program level. The lessons shared by the program leaders at Queen’s University, through their lived experience, will hopefully assist you to get a grip on your CBME change journey.
